# Supplementary material for: The Effect of High Protein Powder Structure on Hydration, Glass Transition, Water Sorption, and Thermomechanical Properties
Source: Foods. 2022 Jan 21;11(3):292. doi: 10.3390/foods11030292 (PMC8834494; doi:10.3390/foods11030292)
Supplement: Supplementary file 1 [file foods-11-00292-s001.zip › foods-1518220-supplementary.pdf]

***Supplementary** material*

S1. Rheology

The apparent viscosity values of MPC solutions at 5, 10, 15 and 20% total solids (Fig. 5) increased with increasing solids contents in all systems, which correlate with previous studies. As expected, the apparent viscosity values decreased upon heating for all non-NI and NI powders. This data was converted to relaxation time – temperature dependence using equations 4 and 5. There is no significant difference for rheological data between dispersions prepared from different MPC powders, due to the same chemical contents of studied systems and given the fact that the powders were fully dispersed and dissociated prior to viscosity measurements.

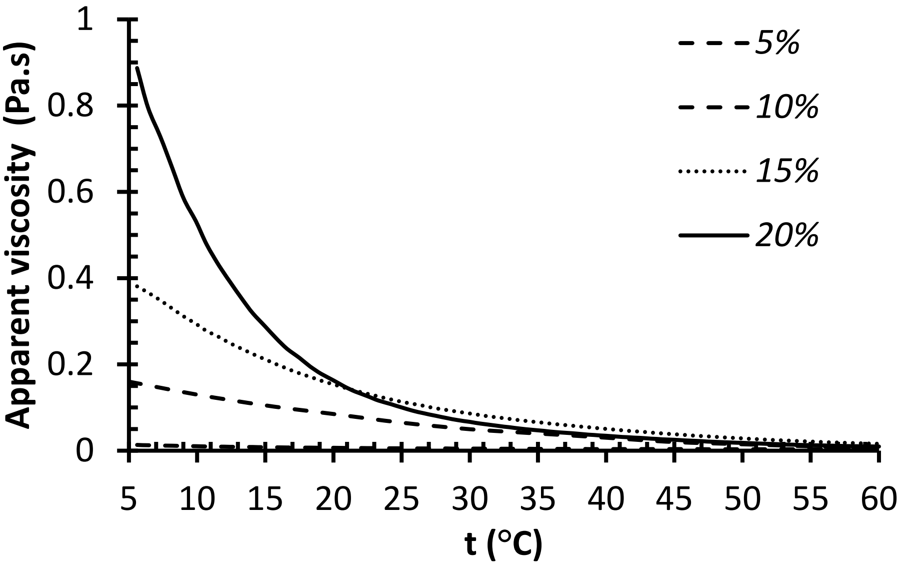

Figure S1. Temperature dependence of apparent viscosity for aqueous milk protein concentrate powder (MPC) dispersions at 5, 10, 15 and 20%, total solids.

Table S1. Glass transition temperatures (T_g) calculated by Gordon-Taylor equation of regular (R), regular NI (RN), agglomerated (A) and agglomerated NI (AN) MPC powders with high water contents.

Water content, %	Glass transition temperature (T_g) of MPC systems, °C			
	R	A	RN	AN
**80**	- 128	- 123	- 128	- 125
**85**	- 130	- 127	- 130	- 128
**90**	- 132	- 130	- 132	- 130
**95**	- 134	- 132	- 134	- 133

Table S2. Structural Strength S (at d = 4) for regular (R), regular NI (RN), agglomerated (A) and agglomerated NI (AN) MPC powders equilibrated at different relative humidity values (RHs).

Relative Humidity	R	A	RN	AN
**RH 0%**	67.0 ± 2.7	66.1 ± 3.5	63.6 ± 2.7*	61.7 ± 2.7*
**RH 23%**	49.7 ± 1.7	46.6 ± 2.7	44.3 ± 4.2*	41.6 ± 2.5*
**RH 33%**	40.7 ± 3.1	39.7 ± 2.3	36.6 ± 3.3*	38.7 ± 1.8
**RH 44%**	38.4 ± 2.1	37.3 ± 1.9	27.9 ± 2.9*	27.4 ± 1.6*

Values presented are the means of triplicate measurements ± standard deviation obtained within the -2 to +2 logarithmic scale. *denotes values of powders significantly different to the control (i.e., R-MPC) powder.
